# An Automatic Multidocument Text Summarization Approach Based on Naïve Bayesian Classifier Using Timestamp Strategy

**DOI:** 10.1155/2016/1784827

**Published:** 2016-02-29

**Authors:** Nedunchelian Ramanujam, Manivannan Kaliappan

**Affiliations:** ^1^Department of Computer Science and Engineering, Sri Venkateswara College of Engineering, Pennalur, Sriperumbudur TK 602117, India; ^2^Department of Information Technology, RMK Engineering College, Kavaraipettai 601206, India

## Abstract

Nowadays, automatic multidocument text summarization systems can successfully retrieve the summary sentences from the input documents. But, it has many limitations such as inaccurate extraction to essential sentences, low coverage, poor coherence among the sentences, and redundancy. This paper introduces a new concept of timestamp approach with Naïve Bayesian Classification approach for multidocument text summarization. The timestamp provides the summary an ordered look, which achieves the coherent looking summary. It extracts the more relevant information from the multiple documents. Here, scoring strategy is also used to calculate the score for the words to obtain the word frequency. The higher linguistic quality is estimated in terms of readability and comprehensibility. In order to show the efficiency of the proposed method, this paper presents the comparison between the proposed methods with the existing MEAD algorithm. The timestamp procedure is also applied on the MEAD algorithm and the results are examined with the proposed method. The results show that the proposed method results in lesser time than the existing MEAD algorithm to execute the summarization process. Moreover, the proposed method results in better precision, recall, and *F*-score than the existing clustering with lexical chaining approach.

## 1. Introduction

Data mining is the domain in which rapid changes are evolved in the recent years due to the enormous advances in the software and hardware technology. The advancement leads to the availability of various kinds of data, which is especially suitable for the instance of text data. The software and hardware platforms used for the social networks and web have facilitated the rapid generation of huge repositories of various types of data. Generally, the structured data are managed with the help of database system, whereas text data are generally managed by search engine due to the lack of structures. The search engine allows the web user to identify the necessary information from the collected works suitably with the help of keyword query. Text summarization is defined as the process of refining the most useful information from the source document to provide an abridged version for the specific task. This paper focuses on developing a multidocument text summarization approach.

Multidocument summarization (MDS) is an automatic process where the essential information is extracted from the multiple input documents. Many approaches are already proposed on retrieving the summary from the single or multiple documents. Single and multiple document summarization approaches have many challenges. The main problem in MDS occurred due to the collection of multiple resources from where the data is extracted, because it may contain the risk of higher redundant information than it is generally found in single document. Moreover, the ordering of extracted information into the coherent text to formulate a coherent summary is a nontrivial task. Summarization can be executed as either abstractive or extractive. Abstractive summarization generally requires information combination, sentence compression, and reformulation. It is complex problem due to the deeper analysis of the input documents and the concept-to-text formulation. Extractive summarization includes assigning saliency factors to some units (paragraph, sentences) of the documents. The sentence extraction is based on the highest score to include in the final summary.

Nowadays, most of the researchers focus their research in automatic text summarization are extractive summarization. Some of the basic extractive processes are as follows:

- Coverage: extraction plays a major role in text summarization process. It recognizes the necessary information that covers the diverse topics in input documents. It can be applied at any level of text paragraphs such as sentence, paragraph, word, and phrase. Numerous algorithms have been proposed to recognize the most important information from the input documents.
- Coherency: optimal ordering of retrieved sentences to formulate the coherent context flow is the complex issue. In single document text summarization, one probable ordering sentence is given by the input text document itself. Still, this process is a nontrivial task. In MDS, the classification is categorized into two approaches: learning the natural sequence of the sentence from the huge corpora and the use of chronological information.
- Redundancy elimination: due to the length limitation needed for an effective summary, and the existence of the extracted sentences which contains the same information, most of the approaches utilize the similarity measure to identify the duplication information from the input documents.

This paper introduces an automatic text summarization approach to overcome the difficulties in the existing summarization approaches. Here, Naïve Bayesian Classification approach is utilized to identify the necessary keywords from the text. Bayes method is machine learning method to estimate the distinguishing keyword features in a text and retrieves the keyword from the input based on this information. The features are generally independent and distributed. Scoring is estimated for the retrieved sentence to compute the word frequency. The combination of this Naïve Bayesian, scoring and timestamp concept helps to improve the summarization accuracy. The proposed summarization method achieves better coverage and coherence using the Naïve Bayesian classifier and the concept of timestamp; hence it automatically eliminates the redundancy in the input documents.

The remainder of this paper is organized as follows.  Section 2 summarizes the related works in the multidocument text summarization.  Section 3 shows the proposed Naïve Bayesian based text summarization approach and the introduction of timestamp strategy.  Section 4 describes the performance analysis. And finally, the paper ends with the conclusion and future work at Section 5.

## 2. Related Works

This section discusses some of the existing MDS approaches. Radev et al. [1] discussed the MEAD algorithm which is the platform for multidocument text summarization. This paper provides the functionality of the MEAD, a public domain, comprehensive, open source, and multidocument multilingual summarization. It has been widely used by more than 500 companies and organizations. It has been applied in various applications ranging from the mobile phone technologies to web page for summarization and also for novelty identification. Ma and Wu [2] combined *n*-gram and dependency word pair for multidocument summarization. The dependency work pair defines the syntactic relationships among the words. Each feature reproduces the cooccurrence of the common topics of multidocuments in diverse perspective. The sentence score was estimated based on the weighted sum of the features. Finally, the summary was formulated by retrieving the salient sentences based on the higher significance score model.

Alguliev et al. [3] designed an evolutionary optimization algorithm for multidocument summarization. This algorithm creates a summary by collecting the salient sentence from the multiple documents. This approach utilizes the summary-to-document collection, sentence-to-document collection, and sentence-to-sentence collection to choose the most important sentences from the multiple documents. Hence, it reduces the redundancy in the document summary. According to the individual fitness value, the algorithm can adaptively alter the crossover rate. The authors in [4] also proposed constraint driven document summarization models. This approach was developed based on the following two constraints: (1) diversity in summarization to reduce the redundancy in the summary among the sentences; (2) sufficient coverage to avoid the loss of document major information to generate the summary. In order to solve the Quadratic Integer Programming (QIP) problem, this approach utilized a discrete Particle Swarm Optimization (PSO) algorithm.

Baruque and Corchado [5] presented a weighted voting summation of Self-Organization Maps (SOMs) ensembles. Weighted voting superposition was used for the outcomes of an ensemble of SOM. The objective of this algorithm was to attain the minimal topographic error in the map. Hence, a weighted voting process was done among the neurons to calculate the properties of the neurons located in the resulting map. Xiong and Lu [6] introduced an approach for multidocument summarization using Latent Semantic Analysis (LSA). Among the existing multidocument summarization approaches, the LSA was a unique concept, which uses the latent semantic information rather than the original features. It has chosen the sentence individually to remove the redundant sentences. Su and Xiaojun [7] discussed an extractive multidocument summarization. This approach uses the semantic role information to improve the graph based ranking algorithm for summarization. The sentence was parsed to obtain the semantic roles. The SRRank algorithm was proposed to rank the sentence, words, and semantic role simultaneously in a heterogeneous ranking manner.

Yang et al. [8] introduced a contextual topic model for multidocument summarization. This method uses the concept of *n*-grams into latent topics to extract the word dependencies placed in the local context of the word. Heu et al. [9] presented a multidocument summarization based on social folksonomy. These approaches use the machine learning and probability based approach to summarize the multiple documents. This approach includes the tag clusters used by a folksonomy, Flickr system to detect the sentences used in multiple documents. A word frequency table was created to examine the contributions and semantics of words with the help of HITS algorithm. The tag clusters were exploited to analyze the semantic link among the words in the frequency table. At last, a summary was created by examining the importance of every word and its semantic relationships to each other.

Glavaš and Šnajder [10] proposed event graphs for information retrieval and multidocument summarization. An event based document representation approach was introduced to filter and structure the details about the events explained in the text. Rule based models and machine learning were integrated to extract the sentence level event and evaluate the temporal relations among them. An information retrieval approach was used to measure the similarity among the documents and queries by estimating the graph kernels across event graphs. Ferreira et al. [11] designed a multidocument summarization model based on linguistic and statistic treatment. This approach extracts the major concern of set of documents to avoid the problems of this kind of summarization such as diversity and information redundancy. It was obtained with the help of clustering algorithm which uses the statistic similarities and linguistic treatment. Meena and Gopalani [12] designed a domain independent framework for automatic text summarization. This method was applied for both the abstractive and extractive text summarization. The source document text was categorized and set of rules are applied for summarization.

Sankarasubramaniam et al. [13] introduced a text summarization using Wikipedia. This approach constructs a bipartite sentence concept graph and the input sentences were ranked based on the iterative updates. Here, a personalized and query focused summarization was considered for user queries and their interests. The Wikipedia based multidocument summarization algorithm was proposed, which permits the real time incremental summarization. Khan et al. [14] presented a framework for multidocument abstractive summarization based on semantic role labelling. Labelling was used for semantic illustration of text. The semantic similarity measure was applied on the source text to cluster the semantically similar sentences. Finally, they were ranked based on the features weighted using Genetic Algorithm (GA). Erkan and Radev [15] proposed a Lexpage rank approach for multidocument text summarization. A sentence connectivity matrix was formulated based on the cosine similarity function. If the cosine similarity among the two sentences goes beyond a specific threshold, then the edge was appended to the connectivity matrix.

Zheng et al. [17] exploited conceptual relations of sentences for multidocument summarization. This concept was composed of three major elements. They were concept clustering, sentence concept semantic relation, and summary generation. The sentence concept semantic relation was attained to formulate the sentence concept graph. The graph weighting algorithm was run to obtain the ranked weighted concepts and sentences. Then, clustering was applied to remove the redundancy and summary generation was conducted to retrieve the informative summary. Celikyilmaz and Hakkani-Tür [18] proposed an extractive summarization. An unsupervised probabilistic model was proposed to formulate the hidden abstract concepts over the documents and also the correlation among the concepts to create topically nonredundant and coherent summaries. The higher linguistic quality was estimated in terms of readability, coherence, and redundancy.

## 3. Materials and Methods: An Automatic Multidocument Text Summarization

### 3.1. Frequent Document Summarization Based on Naïve Bayesian Classifier

Keywords are the important tools which provide the shortest summary of the text document. This paper proposes an automated keyword extraction. It identifies the keywords from the training set, based on their frequencies and positions. In order to extract the summary from the frequently used documents, this paper proposes a Naïve Bayesian classifier in addition to the supervised learning.

#### 3.1.1. Frequent Document Summarization

Consider that the input is the cluster of text documents on similar topic. Here, the task is to extract the frequently used documents and generate a small paragraph which safeguards the major detail contained in the source document. Here, a set of seven multistage compression steps are introduced:

1. Set of documents is provided for processing.
2. From the set of documents, frequently used related documents are selected by the system for processing.
3. Preprocessing work is done and the sentences are broken into words.
4. Score is calculated for each word using Bayesian classifier.
5. Score is calculated for each sentence.
6. For each sentence group, one sentence level illustration is selected.
7. The sentence level illustration is either generated as a linear sentence or further compressed if necessary.

At each stage, this compression process reduces the complexity in the representation using different techniques.

The score value is estimated based on the following equation:

(1) $$\begin{array}{c} \text{Score}\left(\;S_{i}\;\right)=\sum \left(\;wcC_{i,k}+wpP_{i,k}+wfF_{i,k}\;\right), \end{array}$$

where *C*_*i*,*k*_ is centroid value calculated from (2). *P*_*i*,*k*_ is positional value calculated from (3). *F*_*i*,*k*_ is first sentence overlap value calculated from (4). *wc*, *wp*, *wf* are weights. These are the constant values assumed.

The centroid value for sentence *S*_*i*,*k*_ is defined as the normalized sum of the centroid components. It is defined in the following:

(2) $$\begin{array}{c} C_{i,k}=\sum \frac{\left(\text{TF}\left(\alpha_{i}\right)*\text{IDF}\left(\alpha_{i}\right)\right)}{\left|R\right|},\;\alpha \in S_{i,k}, \end{array}$$

where *C*_*i*,*k*_ is normalized sum of the centroid values.

The positional value for the sentence is computed from the following:

(3) $$\begin{array}{c} P_{i,k}=\frac{n-i+1}{n}*C_{\max}, \end{array}$$

where *C*_max⁡_ is the maximum centroid value of the sentence. The overlap value is computed as the inner product of the sentence vectors for the current sentence *i* and the first sentence of the document. The sentence vectors are the *n* dimensional representations of the words in each sentence, whereby the value at position *i* of a sentence vector indicates the number of occurrences of that word in the sentence. The first sentence overlap is calculated from the following:

(4) $$\begin{array}{c} F_{i,k}=\vec{S_{i,k}}*\vec{S_{1,k}}. \end{array}$$

#### 3.1.2. Keyword Extraction

The machine learning algorithms take the keyword extraction as the classification problem. This process identifies the word in the document which belongs to the class of ordinary words or keywords. Bayesian decision theory is the basic statistical technique which depends on the tradeoffs between the classification decisions using the cost and probability that go along with the decisions. It assumes that the problem is given in probabilistic terms and the necessary values are already given. Then it decides on the best class that gives the minimum error with the given example. In cases where there is no distinction made in terms of cost between classes for classification errors, it chooses the class that is most likely with the highest probability.

#### 3.1.3. Preprocessing the Documents

It is a known fact that all the words in the document do not have the same prior chance to be selected as a keyword. Initially, all the tokens are identified with the help of delimiters like tabs, new lines, spaces, dots, and so forth. Moreover, TF*∗*IDF score appears as barrier for such words but may not be sufficient. Hence, it should be removed by assigning the prior probability of zero. Numbers and nonalphanumeric characters also need to be eliminated. After the removal of these words in the document, the model is accurately constructed. It helps to improve the summary generation.

#### 3.1.4. Model Construction

The words in the document should be categorized with the help of keyword properties and features in order to recognize whether the word is labeled as the key or not. The word frequency is estimated based on the number of times the word appears in the text. Generally, prepositions have no value as the keyword. If a word has higher frequency than the other documents, this can be another categorizing feature to decide the word is key or not. Combining the above two properties, the metric TF*∗*IDF is computed. TF represents the term frequency and IDF represents the inverse document frequency. It is the standard measure used for information extraction [19].

Word *α* in document *ℛ* is defined as follows:

(5) TF∗IDFP,R=Pword  in  R  is  α∗−log⁡Pα  in  a  document.

(i) First term in this equation: it is estimated by computing the number of times the word placed in the document and splitting it to the total number of words.
(ii) Second term in this equation: it is estimated by counting the total number of documents in the training set, where the word occurs except *ℛ*, and dividing the value by the total number of documents in the training set.

Another feature is the* position* of the word places in the document. There are many options to characterize the position. The first property is the word distance to the beginning of the text. Generally, the keyword is concerted in the beginning and end of the text. Sometimes, the more essential keywords are identified in the beginning or end of the sentence. Bayes theorem is applied to compute the probability that the word is the keyword and it is defined as follows:

(6) Pkey ∣ T,R,PT,PS=PT ∣ key∗PR ∣ key∗PPT ∣ key∗PPS ∣ keyPT,R,PT,PS.

Notations section describes the list of notations.

### 3.2. Visiting Time Examination

Algorithms 1 and 2  are used to calculate the visiting time for each document and also for selecting the document for processing.

### 3.3. Summary Generation

After performing the Naïve Bayesian operation the summary generation is carried out based on Score and applying timestamp.  Algorithm 3 which is for score and timestamp calculation is discussed in the following sections.

#### 3.3.1. Summary Generation Based on Score

The final summary is generated based on the score. After processing the documents by using the Naïve Bayesian, the sentences are arranged based on the score. High score sentence will appear first, next high score sentence, and so on. The summary generated by Naïve Bayesian includes the selected sentences for each document and result them in the order in the source document. It should be noted that sentence present from the first document will be placed before the sentences selected from the next document subsequently. The sequence of the sentences in the document summary may not be reasonable in occurrence. In order to overcome this issue, the timestamp strategy is implemented.

#### 3.3.2. Summary Generation Based on Timestamp

The timestamp strategy is implemented by assigning a value to every sentence of the document. It is based on the chronological position of the document. Once the sentences are chosen, immediately they are arranged in the ascending order based on the timestamp. An ordered look helps to achieve the summary, which carries out a coherent looking summary. The total number of sentences in the summary is represented by the compression rate.

## 4. Results and Discussion

This section discussed the experimental results obtained for the proposed Naïve Bayesian based multidocument summarization and the comparative results with the MEAD algorithm. The timestamp procedure is also applied on the MEAD algorithm and the results are examined with the proposed method. There are totally 20 input documents which are taken for analysis and the inputs are processed. Some of the input documents are the following.

*Input Document 1*

1. Born in Mumbai (then Bombay) into a middle-class family, Sachin Tendulkar was named after his family's favorite music director Sachin Dev Burman.
2. Sachin Tendulkar went to Sharadashram Vidyamandir School where he started his cricketing career under coach Ramakant Achrekar.
3. While at school, Sachin Tendulkar was involved in a mammoth 664 run partnership in a Harris Shield game with friend and team mate Vinod Kambli.
4. In 1988/1989, Sachin Tendulkar scored 100 not-out, in his debut first-class match, for Bombay against Gujarat.
5. At 15 years and 232 days Sachin Tendulkar was the youngest to score a century on debut.

*Input Document 2*

1. Sachin is the fastest to score 10,000 runs in Test cricket history, He holds this record along with Brian Lara, Both of them achieved this feat in 195 innings.
2. Sachin scored 4th highest tally of runs in Test cricket (10,323).
3. Career Average 55.79—Sachin has the highest average among those who have scored over 10,000 Test runs.
4. Tendulkar is the second Indian to make over 10,000 runs in Test matches.
5. Sachin has 37 Test wickets (14 Dec 2005).
6. Sachin is the second fastest player to reach 9000 runs (Brian Lara made 9000 in 177 innings, Sachin in 179.).

*Input Document 3*

(1) Sachin has highest batting average among batsmen with over 10,000 ODI runs (as of March 17, 2006).
(2) Sachin has scored highest individual score among Indian batsmen (186^*∗*^ against New Zealand at Hyderabad in 1999).
(3) Sachin holds the record for scoring 1,000 ODI runs in a calendar year, He has done it six times—1994, 1996, 1997, 1998, 2000 and 2003.
(4) In 1998 Sachin made 1,894 ODI runs, still the record for ODI runs by any batsman in any given calendar year.
(5) In 1998 Sachin hit 9 ODI centuries, the highest by any player in a year.

*Input Document 4*

1. Sachin's first ODI century came on September 9, 1994 against Australia in Sri Lanka at Colombo. It had taken Tendulkar 79 ODIs to score a century.
2. Sachin Tendulkar is the only player to score a century while making his Ranji Trophy, Duleep Trophy and Irani Trophy debut.
3. Wisden named Tendulkar as one of the Cricketers of the Year in 1997, the first calendar year in which he scored 1,000 Test runs.

### 4.1. Input Documents and Number of Times Visited

Automatic process has been developed to monitor and calculate the number of times the document has been read by the users. Input documents along with the number of times visited are calculated and the results are given in Table 1.

### 4.2. Frequent Documents Summarization

Instead of taking up each sentence for comparison for summarization from all documents, it would be more than enough to summarize only the document which has been put to many numbers of readers. Since we track the document which is read frequently by many people, it is supposed to provide all the necessary information about the topic to the user so the user need not surf through other documents for information as the document in hand would be satisfactory.

#### 4.2.1. Frequent Documents Selected for Processing

In the total of 20 documents, we have selected 10% (2 documents) of documents as frequently used documents for processing. Since the Input 6.doc and Input 7.doc are visited more number of times they have been considered for further analysis. It is shown in Table 2.

### 4.3. Summary Generation

**Figure 1:**
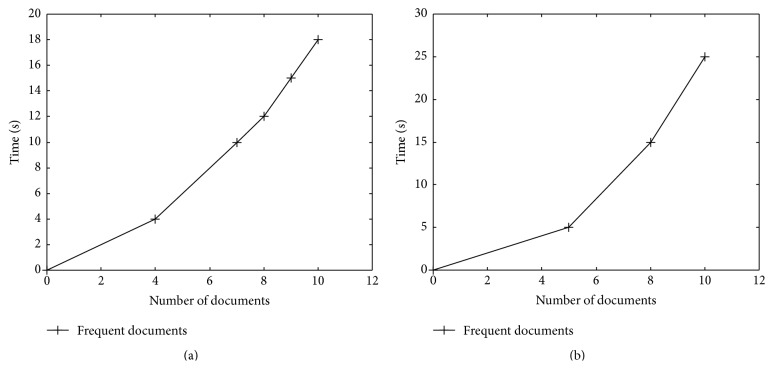
Frequent documents summarization: (a) Naïve Bayesian classifier (proposed) and (b) MEAD (existing).

The performance for summarization of the input documents using Naïve Bayesian classifier and MEAD has been analyzed and compared with frequent documents using Naïve Bayesian classifier and MEAD. In total there are 100 documents; among them 10% of documents are selected as frequent documents. The performance graph for frequent document summarization is shown in Figure 1. From the graph, it visually shows that the proposed Naïve Bayesian takes lesser time than the existing MEAD algorithm.

### 4.4. Score Table for Multidocuments

**Figure 2:**
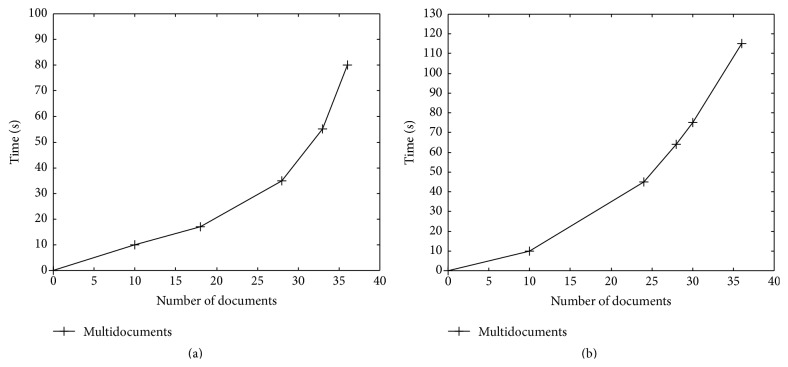
Multidocument summarization: (a) Naïve Bayesian classifier (proposed) and (b) MEAD (existing).

From Figure 2(a), it is understood that when the Naïve Bayesian classifier is applied on the multidocuments the time taken to get the summary is 80 sec which is higher than the time taken to summarize the frequent documents only. From Figure 2(b), it is understood that when the MEAD is applied on the multidocuments the time taken to get the summary is 115 sec which is higher than the time taken to summarize the frequent documents only. The proposed Naïve Bayesian classifier takes 80 sec which is lesser than the MEAD algorithm.

**Figure 3:**
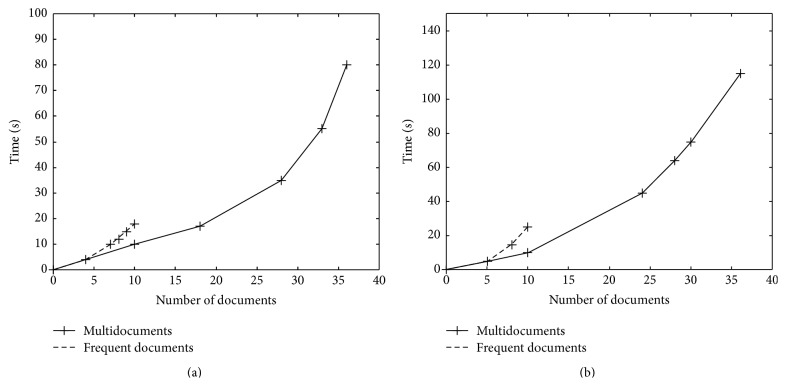
Comparison of frequent versus multidocument summarization: (a) Naïve Bayesian classifier (proposed) and (b) MEAD (existing).

Figure 3 shows the comparison of frequent and multidocument summarization using MEAD and Naïve Bayesian classifier. Comparison of frequent and multidocument summarization using MEAD and Naïve Bayesian classifier shows that when the Naïve Bayesian classifier is applied on the multidocuments the time taken to get the summary is 80 sec and the number of documents is 36 which is less than the time taken by MEAD where the time taken is 115 sec for 36 documents. Also for summarizing only the 10 frequent documents the Naïve Bayesian classifier takes only 18 sec which is less than the time taken by MEAD where the time taken is 25 sec for 10 documents. So it is proved that summarization of frequent documents using Naïve Bayesian classifier is better when compared to MEAD.

Table 3 compares the run time taken to summarize the documents using the two techniques. From the table it is observed that the proposed Naïve Bayesian classifier results in lesser time than the existing MEAD algorithm.

The performance measures such as precision, recall, and *F*-score are calculated for the proposed method and compared with the existing method [16]. Precision estimates the correctness percentage and recall measures the completeness of the summarizer. Precision also is used to estimate the usefulness of the summarizer and recall is used to show effectiveness of the model. With the combination of precision and recall, *F*-score is evaluated. The evaluation measures are computed based on the following equation:

(7) precision=relevant  sentences∩retrieved  sentencesretrieved sentences,recall=relevant sentences∩retrieved sentencesrelevant sentences,F-Score=2∗precision∗recallprecision+recall.

Table 4 shows the comparison between the proposed method and the existing method. From the results, it is evidently proved that the proposed method works better than the existing method.

### 4.5. Evaluation of the Summary

Multidocument summarization is carried out using MEAD algorithm and Naïve Bayesian classifier for frequent documents with timestamp. This is compared with human generated summary by human assessors consisting of 5 professors, 9 lecturers, and 6 students to find out whether the output is an informative summary, which can be a substitute of the original documents. In order to satisfy this, the following points are considered important and are assessed by human assessors.

*Comprehensibility*. The summary should include main content of target documents exhaustively.

*Readability*. The summary should be a self-contained document and should be readable.

*Length of Summary to Be Generated*. The length of summary generated by human and the system is compared.

Each participant generated summaries according to topic information given and submitted a set of summaries. Each topic corresponds to one IR result, which consists of the following information:

- topic ID,
- list of keywords for query in IR,
- brief description of the information needs,
- set of documents IDs, which are target documents of summarization: the number of documents varies from 3 to 20 according to the topic,
- length of summary to be generated: there are two lengths of summary, “Long” and “Short.” The length of “Long” is twice of “Short,”
- comprehensibility: the summary has all the important content or not,
- readability: the summary is easy to read or not.

#### 4.5.1. Comparative Analysis of Summary Generated by System and Human

About 20 human assessors are used to carry out the comparative study of summary generated by system and human in terms of comprehensibility, readability, and length of the summary.  Table 5 provides information about comparison of length of summary by human assessors and system.

From Table 5 it is observed that the summary generated by the system is shorter than summary produced by human as 70% of the human assessors stated that the length of the summary generated by the system is short.

From Table 6, it is observed that the summary generated by the system has all important contents as 90% of the human assessors stated that the summary generated by the system is comprehensible.

From Table 7, it is observed that the summary generated by the system is easy to read as 100% of the human assessors stated that the summary generated by the system is readable.

From this analysis done using human assessors it is proved that the summary generated by the system is short and the quality of the summary generated is also good based on the two factors readability and comprehensibility.

## 5. Conclusion and Future Work

In this paper, an automatic text summarization approach is proposed which uses the Naïve Bayesian Classification with the timestamp concept. This summarizer works on a wide variety of domains varying between international news, politics, sports, entertainment, and so on. Another useful feature is that the length of the summary can be adapted to the user's needs as can the number of articles to be summarized. The compression rate can be specified by the user so that he can choose the amount of information he wants to imbibe from the documents. To show the system efficiency, it is compared with the existing MEAD algorithm. The results show that the proposed method yields better outputs than the MEAD algorithm. The proposed method results in better precision, recall, and *F*-score than the existing clustering and lexical chaining method.

The proposed work does not involve a knowledge base and therefore can be used to summarize articles from fields as diverse as politics, sports, current affairs, and finance. However, it does cause a tradeoff between domain independence and a knowledge based summary which would provide data in a form more easily understandable to the human user. A possible application of this work can be made to make data available on the move on a mobile network by even shortening the sentences produced by our algorithm and then shortening it. Various NLP based algorithms can be used to achieve this objective. Thus we would first produce a summary by sentence extraction from various documents and then abstractive methods are employed to shorten those sentences produced. This will ensure that the summary produced is to the highest condensed form which can be made in the mobile industry.

## Figures and Tables

**Algorithm 1:**
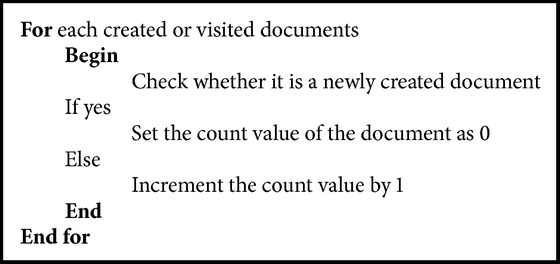
For counting the visiting time of the document.

**Algorithm 2:**
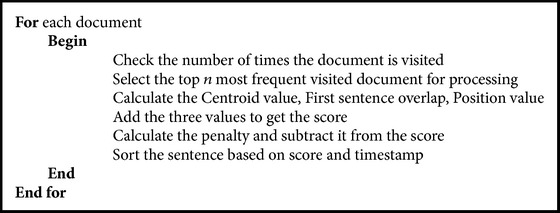
For selecting frequent document for processing.

**Algorithm 3:**
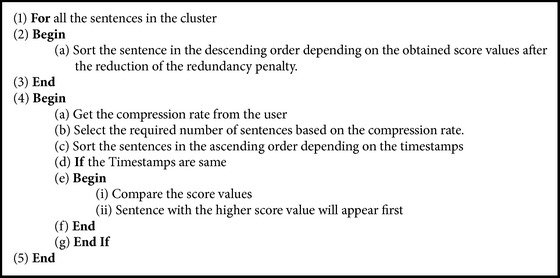
Timestamp based summary generation.

**Table 1:** Input documents and number of times visited.

Documents name	Number of times visited
Input 1.doc	11
Input 2.doc	8
Input 3.doc	7
Input 4.doc	9
Input 5.doc	11
Input 6.doc	12
Input 7.doc	12
Input 8.doc	7
Input 9.doc	10
Input 10.doc	9
Input 11.doc	8
Input 12.doc	5
Input 13.doc	6
Input 14.doc	5
Input 15.doc	10
Input 16.doc	4
Input 17.doc	11
Input 18.doc	8
Input 19.doc	10
Input 20.doc	7

**Table 2:** Frequently visited documents.

Document name	Number of times visited
Input 6.doc	12
Input 7.doc	12

**Table 3:** Comparison of frequent versus multidocument summarization using MEAD and NAÏVE Bayesian classifier.

Summarization techniques	Multidocument time in seconds	Frequent document time in seconds
MEAD	115	25
Naïve Bayesian classifier	80	18

**Table 4:** Comparison of proposed Naïve Bayesian classifier and existing method [16].

Metrics	Proposed method	Existing method
Precision	85.4	78
Recall	83.9	77.7
*F*-measure	92	86

**Table 5:** Comparison of length of summary generated by human and system.

	Length of summary generated by system	
	Short	Long
Professor	3	2
Lecturers	6	3
Students	5	1

**Table 6:** Evaluating the comprehensibility of summary generated by system.

	Comprehensibility	
	Yes	No
Professor	5	Nil
Lecturers	9	Nil
Students	4	2

**Table 7:** Evaluating the readability of summary generated by system.

	Readability	
	Yes	No
Professor	6	Nil
Lecturers	9	Nil
Students	4	Nil

## Notations

TF*∗*IDF score (*T*)*⁡*: Distance to the beginning of the paragraph (*ℛ*)
PT: Relative position of the word with respect to the entire text
PS: Sentence which exists in the entire text
*P*(key)*⁡*: Prior probability that the word is a keyword
*P*(*T*∣key)*⁡*: Probability of TF*∗*IDF score *T* given the word is the keyword
*P*(*ℛ*∣key)*⁡*: Probability of having the neighbor distance *ℛ*
*P*(PT∣key)*⁡*: Probability of having the relative distance
*P*(PS∣key)*⁡*: Probability of having relative distance *ℛ* to the beginning of the paragraph
*P*(*T*, *ℛ*, PT, PS)*⁡*: Probability that a word have TF*∗*IDF score *T*
*S*_*i*,*k*_*⁡*: *i*th sentence in the document
*ℛ⁡*: Document
*C*_max⁡_*⁡*: The maximum centroid value of the sentence
TF: Term frequency
IDF: Inverse document frequency.
